# Acceptance of a Virtual Reality Headset Designed for Fall Prevention in Older Adults: Questionnaire Study

**DOI:** 10.2196/20691

**Published:** 2020-12-14

**Authors:** Nicolas Mascret, Lisa Delbes, Amélie Voron, Jean-Jacques Temprado, Gilles Montagne

**Affiliations:** 1 Aix Marseille Univ, CNRS, ISM Marseille France

**Keywords:** technology acceptance model, acceptability, acceptance, virtual reality, elderly, fall, eHealth, self-efficacy, achievement goals

## Abstract

**Background:**

Falls are a common phenomenon among people aged 65 and older and affect older adults’ health, quality of life, and autonomy. Technology-based intervention programs are designed to prevent the occurrence of falls and their effectiveness often surpasses that of more conventional programs. However, to be effective, these programs must first be accepted by seniors.

**Objective:**

Based on the technology acceptance model, this study aimed to examine the acceptance among older adults before a first use of a virtual reality headset (VRH) used in an intervention program designed to prevent falls.

**Methods:**

A sample of 271 French older adults (mean age 73.69 years, SD 6.37 years) voluntarily and anonymously filled out a questionnaire containing the focal constructs (perceived usefulness, perceived enjoyment, perceived ease of use, intention to use, fall-related self-efficacy, and self-avoidance goals) adapted to the VRH, which was designed to prevent falls.

**Results:**

The results of the structural equation modeling analysis showed that intention to use the VRH was positively predicted by perceived usefulness, perceived enjoyment, and perceived ease of use. Perceived usefulness of the VRH was also negatively predicted by fall-related self-efficacy (ie, the perceived level of confidence of an individual when performing daily activities without falling) and positively predicted by self-avoidance goals (ie, participating in a physical activity to avoid physical regression).

**Conclusions:**

A better understanding of the initial acceptance among older adults of this VRH is the first step to involving older adults in intervention programs designed to prevent falls using this kind of device.

## Introduction

### Background

The number and proportion of people aged 65 years and older in the general population is likely to increase in the coming decades [1]. Among the numerous factors that may influence the quality of life and autonomy of older adults, falls are one of the most frequent and dramatic. Indeed, approximately 40% of people older than 65 years fall each year, about 2.5% of them will be hospitalized, and only one-half of those hospitalized will survive 1 year [2]. Consequently, research on fall prevention is of great scientific, practical, and socioeconomic interest, with an aim to help older adults to live longer independently as well as to reduce the burden on the health care system [3].

Improving gait and postural control through physical activity training interventions has been shown to be effective for preventing falls [4,5]. Novel applications of technology that promote these interventions appear promising. A number of recent reviews and meta-analyses revealed that new technologies (eg, virtual reality [VR], augmented reality, exergames, and artificial intelligence) have opened the door to a new generation of intervention programs designed to prevent the occurrence of falls [6-8]. Recent studies also demonstrated the effectiveness of training programs implemented via new technologies for improving the control mechanisms involved in balance [9,10] and goal-directed locomotion [11,12] in older adults. For instance, specific exergames have been designed to train balance control, focusing on either the static control of the center of mass or its control while performing precise, rapid, and well-directed steps in balance-threatening situations [13,14]. They allow the cognitive and motor demands of the training conditions to be finely controlled during balance control tasks. However, falls most often occur during everyday walking in complex environments. Therefore, interventions that combine gait training and virtual environments strewn with obstacles have been suggested to be more effective for decreasing fall risk than either classic or balance training interventions delivered via commercial exergames [15].

These programs based on virtual environments provide more realistic/ecological stimulations of physical, cognitive, and sensory resources [16], thereby helping to improve adaptive behavior and prevent falls during daily living tasks [11,12,17]. Even if they pursue the same goal (ie, the development of adaptive behaviors), these programs differ from each other in several aspects, including the technological supports (eg, VR helmet versus projection screen), proposed procedure (more or fewer trials distributed over a longer or shorter period), and tasks used to optimize adaptive capacities [18]. It is therefore not surprising that the results obtained in these studies are sometimes different or even contradictory, and it’s often not possible to determine the precise cause of the conflicting results. One explanation for these discrepancies could lie in the different levels of acceptance of the prevention programs. Despite their intrinsic value, as previously identified, technology-based intervention programs must first be accepted by older adults because if they do not see the need for a technology or recognize its usefulness, they are highly unlikely to start using it [19] and may in turn refuse to commit to the training program. Moreover, they may still participate in the program but with less confidence and interest and more psychological discomfort because of their difficulty in accepting the technology to be used. Thus, in view of the potential effectiveness of VR as a rehabilitation tool for fall prevention, it is important to ensure that this tool is well accepted by the target population of the training interventions. This idea has been confirmed and emphasized by a number of recent meta-analyses and reviews [8,20]. Accordingly, the aim of this study was to assess the acceptance of a VR headset (VRH; eg, HTC Vive [HTC Corporation], Oculus Rift [Facebook Technologies, LLC]) by older adults who will use it during a fall-prevention training program. The study of acceptance is thus considered a prerequisite, and will make it possible to refine the analysis of the effectiveness of training interventions, in particular by comparing respondents and nonrespondents.

### Acceptance of Technology and the VRH

**Figure 1:**
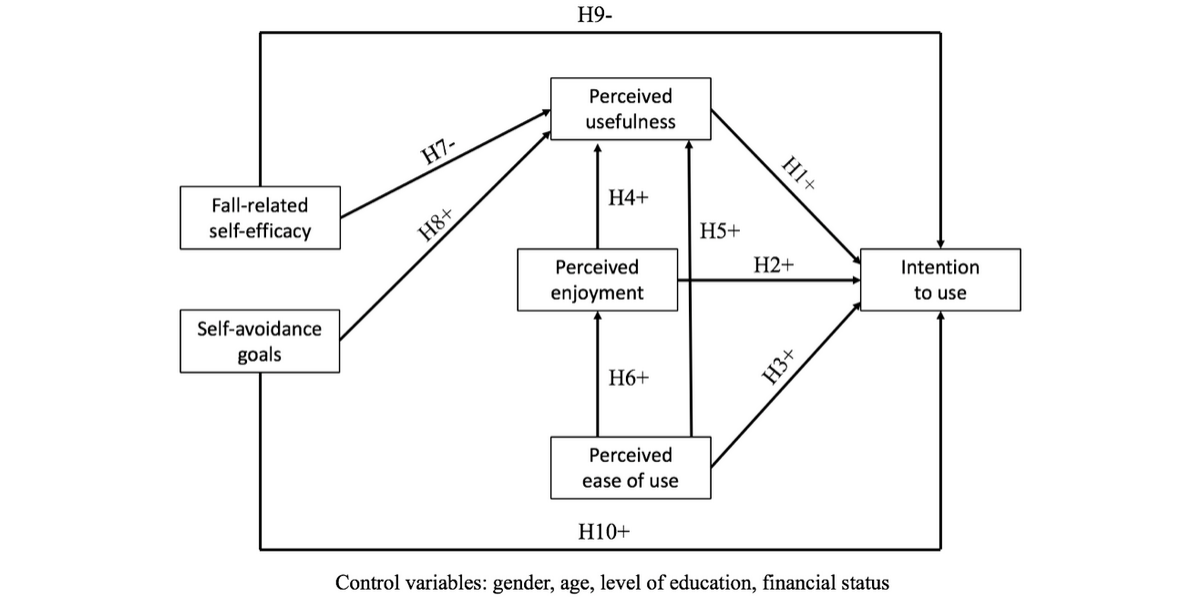
The hypothesized model in the study.

Acceptance of a particular technology may be defined as the psychological determinants of the behavioral intention to use the technology without ever having experienced it and/or after its actual use [21,22]. Acceptance is based on two well-known models in the social psychology literature, namely the theory of reasoned action and the theory of planned behavior, both of which postulate that behavioral intention and effective behavior are determined by attitudes and representations [23,24]. In other words, both the intention to participate and the effective participation in a technology-based training program may be determined by attitudes and representations toward the training program itself, but also toward the technology used in the training program. If attitudes and representations toward the technology are negative, there is a high probability that individuals will not participate in the training program based on this technology despite its potential and validated benefits. In line with these theories, the technology acceptance model (TAM) [25-27] is the most frequently used theoretical framework to study acceptance of technologies in general and of eHealth technologies in particular [28]. The TAM has highlighted that perceived usefulness of a technology and its perceived ease of use are positive predictors of behavioral intention to use the technology, which is itself a predictor of its actual use [25-27]. Perceived enjoyment may also contribute to the acceptance of technology [29]. This last factor is particularly relevant when examining acceptance of VR technologies, which are often considered hedonic [30], especially when they are being used to prevent fall occurrence among older adults [31]. The four components of the TAM (perceived usefulness, perceived ease of use, perceived enjoyment, and intention to use) and their relationships are represented in Figure 1.

However, studies using the TAM to examine acceptance of immersive VR are not very frequent [32], and those that focus on acceptance of VRHs by older adults are even more rare. For instance, acceptance of VRHs by elderly people was considered neutral before use (neither positive nor negative) but became positive after a first use, as compared with a control group of elderly people exposed to time-lapse videos presented on a computer [33]. Older adults showed high acceptance of VRHs after using the devices for 6 weeks, while perceived usefulness, perceived ease of use, and perceived enjoyment had positive effects on the intention to use VRHs [34]. The same pattern of results was found with qualitative investigations and focus groups [35]. However, to the best of our knowledge, acceptance before a first use of a VRH designed to reduce older adults’ risk of falling through their use in intervention programs has not yet been studied. The fact that the positive influence on fall prevention of training programs using VRHs has been validated [10] reinforces the relevance of studying acceptance of such a VRH. Indeed, older adults who have a low acceptance score also have a very high probability of never adopting this kind of VRH despite its objective usefulness to maintain and improve their functional capacities, but this hypothesis has not yet been tested. This study is a step in this direction. The aim of this study was to examine if the TAM variables predicted older adults’ intention to use a VRH in an intervention program designed to prevent falls.

Although the TAM is a validated and widely used model, it often needs to be extended and upgraded to increase its explanatory power in eHealth [21,28]. One of the possibilities highlighted by Venkatesh et al [36] was to examine external variables of interest (ie, antecedents) that may influence perceived usefulness, perceived ease of use, and perceived enjoyment. This was the second aim of our study. Fall-related self-efficacy and self-avoidance goals were two promising psychological variables to investigate because they may be related to older adults’ investment in exercise intervention programs intended to prevent falls.

### Fall-Related Self-Efficacy and Self-Avoidance Goals

Identifying elderly people’s fear of falling is worthwhile to optimize prevention because interventions targeted toward older adults with high levels of fear of falling may help decrease the risk that a fall will occur [37]. Moreover, older people often compensate for their fear of falling by being less physically active (ie, reducing frequency and duration of mobility) [38], which could paradoxically limit their involvement in technology-based training programs intended to prevent fall occurrence. Fear of falling may be assessed by fall-related self-efficacy, which is the perceived level of confidence that an individual experiences while performing several activities of daily living without falling, such as taking a bath or getting out of bed [39,40]. The general construct of self-efficacy has already been used with an elderly sample as an antecedent of the main variables of the TAM (eg, gerontechnology self-efficacy [41]), but to date, fall-related self-efficacy has not been used as an external variable with the potential to influence the main variables of the TAM. However, this variable seems to be relevant here because this study focuses on older adults for whom fall-related self-efficacy tends to decrease with age [39] and because we examine the acceptance of a VRH whose purpose is precisely to reduce the risk of falling through its use in intervention programs. We expected fall-related self-efficacy to be a negative predictor of perceived usefulness and intention to use this VRH. This expected result would highlight that elderly people with lower fall-related self-efficacy are more likely to find this VRH useful and to have the intention to use it because the purpose of this VRH addresses their concerns about fear of falling.

From this perspective, another psychological variable, namely self-avoidance goals, was of interest. Among several achievement goals that may be pursued in the physical activity domain, self-avoidance goals focus on participating in a physical activity to avoid physical regression [42]. In the sport context, self-avoidance goals are prevalent among older athletes on the downside of their careers [42], but these kinds of goals are also overrepresented in older adults whatever the context because elderly individuals expect and encounter more losses in resources than gains [43]. Consequently, maintaining and preserving their current resources is often the main goal of older adults, especially in life domains such as health and physical activity, in which resource depletion may be easily seen [44]. Adopting self-avoidance goals in the physical domain (ie, striving to avoid physical regression) may have a protective role in fighting the effects of aging because it may encourage older adults to be physically active to maintain their level of physical fitness, which may therefore have many positive consequences on their health [45]. Thus, to avoid physical decline, older adults with self-avoidance goals will certainly have more desire to participate in training programs intended to prevent fall occurrence. While some theories of motivation have already been used with the TAM (eg, self-determination theory [46]), achievement goal theory has not yet been investigated. However, endorsing self-avoidance goals may have a positive influence on the adoption of eHealth technologies because the purpose of these technologies may match with the purpose of older adults’ self-avoidance goals (ie, avoiding physical regression). This assumption remains speculative, but we may reasonably believe that older adults’ self-avoidance goals in the physical domain could be positive predictors of the perceived usefulness and intention to use a VRH designed to reduce older adults’ risk of falling through its use in intervention programs.

### Objectives and Hypotheses

To the best of our knowledge, among the few studies that have investigated the acceptance of VRHs in older adults, no study has focused on the use of this technological device for fall prevention [33,34]. Thus, the first aim of this study based on the TAM was to examine older adults’ acceptance of a VRH specifically designed to reduce older adults’ risk of falling through its use in intervention programs. The second aim of the study was to investigate the potential predictive role of two psychological variables (fall-related self-efficacy and self-avoidance goals) on the perceived usefulness and intention to use such a device. These variables have not yet been used to extend the TAM, but they appear to be relevant in the context of eHealth technologies designed for fall prevention.

Based on the previous literature review focusing on the TAM, we first hypothesized that older adults’ intention to use a VRH specifically designed for fall prevention would be positively predicted by perceived usefulness (H1), perceived enjoyment (H2), and perceived ease of use (H3). Moreover, we expected perceived usefulness to be positively predicted by perceived enjoyment (H4) and perceived ease of use (H5). We also expected perceived enjoyment to be positively predicted by perceived ease of use (H6). Based on the literature reviews focusing on fall-related self-efficacy and self-avoidance goals, we hypothesized that perceived usefulness would be negatively predicted by fall-related self-efficacy (H7) and positively predicted by self-avoidance goals (H8). Finally, we expected intention to use to be negatively predicted by fall-related self-efficacy (H9) and positively predicted by self-avoidance goals (H10). All hypotheses and the model tested in the present study are summarized in Figure 1.

## Methods

### Participants and Procedure

The study sample included 271 French volunteers (171 women, 100 men) aged 65 to 84 years (mean age 73.69 years, SD 6.37 years). Participants were recruited during the last 3 months of 2019 and met the following eligibility criteria: (1) aged 65 years or older, (2) able to walk without a walking aid, and (3) had never used a VRH before. They voluntarily and anonymously filled out the questionnaire containing focal constructs and demographic information that may influence acceptance of VRH (gender, age, level of education, and financial status). Before completing the questionnaire and following the procedure frequently used with older adults [47], participants had to read a short text accompanied by photos. The short text described a VRH (ie, a helmet-mounted display that completely covers the eyes for an immersive 3-dimensional experience) and its specific purpose in the study (ie, to prevent older adults’ falls through its use in exercise intervention programs). Five photos were included beside the text showing a VRH alone, a man wearing a VRH, a virtual scene, an elderly lady in a virtual scene wearing a VRH, and an older man in another virtual scene. This procedure was used because many older adults do not necessarily know what a VRH looks like or what it may be used for.

The questionnaire was completed in individual paper-based sessions. Volunteers gave their consent and were given the opportunity to stop their participation at any time during the study without any consequences. The study was conducted in accordance with the Declaration of Helsinki and met the requirements of the Commission Nationale de l’Informatique et des Libertés (n 2004-801). Table 1 summarizes the participants’ demographic characteristics.

**Table 1:** Participants’ demographic characteristics (N=271).

Characteristics		Values, n (%)
**Gender**		
	Women	171 (63.1)
	Men	100 (36.9)
**Age (years)**		
	65-74	164 (60.5)
	75-84	90 (33.2)
	≥85	17 (6.3)
**Level of education**		
	No diploma	18 (6.6)
	Low-level diploma	71 (26.2)
	Medium-level diploma	62 (22.9)
	High-level diploma	27 (10.0)
	Short graduate diploma	40 (14.8)
	Long graduate diploma	53 (19.6)
**Financial status**		
	Adequate financial resources	102 (37.6)
	Adequate financial resources, except for unforeseen circumstances	80 (29.5)
	Scarce financial resources	53 (19.6)
	Lack funding	36 (13.3)

### Measures

Perceived usefulness, perceived ease of use, perceived enjoyment, and intention to use were assessed through a total of 12 items focusing on the VRH that was designed to reduce older adults’ risk of falling through physical training. These items were adapted from two studies focusing on the acceptance of technology to improve health [48] and on the acceptance of VR hardware [30]. Participants responded to the 3 items used for each variable on a Likert scale ranging from 1 (strongly disagree) to 5 (strongly agree). Actual use, which is a component of the TAM [25], was not investigated because actual use was an exclusion criterion in this study. Instead, we wanted to examine the behavioral intention to use a VRH for preventing falls without having experienced the device.

Fall-related self-efficacy was assessed with the 14-item Modified Falls Efficacy Scale [39], validated in French [40]. Participants rated their level of confidence in performing several activities of daily living (eg, getting dressed and undressed, going up or down stairs) without falling on an 11-point scale ranging from 0 (not at all confident) to 10 (completely confident). The mean of all 14 items represents the fall-related self-efficacy score, with a higher score indicating greater confidence.

Self-avoidance goals were assessed with the corresponding subscale of the 3×2 Achievement Goal Questionnaire for Sport [42], adapted to physical activity. Participants answered the 3 items (eg, “When I do physical activity, my goal is to avoid doing worse than I usually do”) on a Likert scale ranging from 1 (strongly disagree) to 5 (strongly agree).

Internal consistency was good for each variable, with McDonald omegas ranging from 0.89 to 0.96. McDonald omegas were used instead of Cronbach alphas because the latter have the tendency to over- or underestimate reliability [49]. McDonald omegas for each variable, descriptive statistics, and the complete list of items are provided in Table 2.

**Figure 2:**
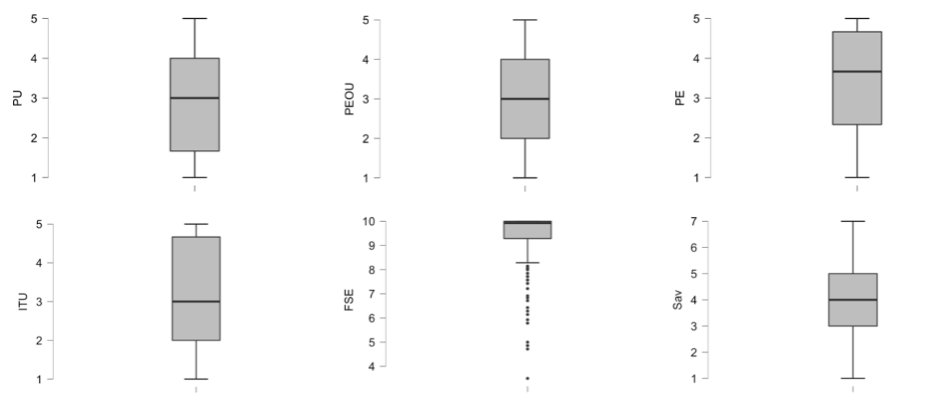
Boxplots for all variables. FSE: fall-related self-efficacy; ITU: intention to use; PE: perceived enjoyment; PEOU: perceived ease of use; PU: perceived usefulness; Sav: self-avoidance goals.

Most of the scales’ standard deviations were found to be very high, except for fall-related self-efficacy. Figure 2 shows the boxplot for each variable, highlighting that participants’ responses on items assessing perceived usefulness, perceived ease of use, perceived enjoyment, intention to use, and self-avoidance goals were scattered across the entire Likert scale.

**Table 2:** Descriptive statistics, internal consistency, and items for each variable.

Variables		Mean (SD)	McDonald omega
**Perceived usefulness**		2.80 (1.25)	0.95
	I believe using this VRH would be useful for improving my health.		
	I believe using this VRH would enable me to improve my health.		
	I believe using this VRH would help me improve my health.		
**Perceived ease of use**		3.10 (1.27)	0.92
	I believe using this VRH would be clear and understandable.		
	I would find this VRH easy to use.		
	I believe using this VRH would be easy for me.		
**Perceived enjoyment**		3.42 (1.39)	0.93
	I believe I would find using this VRH enjoyable.		
	I believe I would have fun using this VRH.		
	I believe I would find using this VRH exciting.		
**Intention to use**		3.15 (1.42)	0.95
	Assuming I had access to this VRH, I would like to use it.		
	Assuming I had access to this VRH, I would intend to use it.		
	Assuming I had access to this VRH, I predict that I would use it.		
**Fall-related self-efficacy**		9.41 (1.07)	0.96
	Getting dressed and undressed/preparing a simple meal/taking a bath or a shower/getting in or out of a chair/getting in or out of bed/answering the door or telephone/walking around the inside of the house/reaching into cabinets or closets/light housekeeping/simple shopping/using public transportation/crossing roads/light gardening or hanging out the washing/going up or down stairs		
**Self-avoidance goals**		3.57 (1.39)	0.89
	When I do physical activity, my goal is to avoid having worse results than I had previously.		
	When I do physical activity, my goal is to avoid doing worse than I usually do.		
	When I do physical activity, my goal is to avoid being less effective compared with my usual level of performance.		

### Data Analysis

To evaluate the model’s fit [50,51], we used the χ^2^/df ratio (value ≤3), the comparative fit index (CFI; value ≥0.90), the Tucker-Lewis index (TLI; value ≥0.90), the root mean square error of approximation (RMSEA; value ≤0.08), and the standardized root mean square residual (SRMR; value ≤0.08). A structural equation modeling (SEM) analysis using the maximum likelihood estimations [52] was conducted to test the previous hypotheses (see Figure 1) with the JASP software (version 0.10). Gender, age, level of education, and financial status were entered into the model to control for these variables. According to Kline [53], a typical sample size in studies using SEM is approximately 200 participants. This study met the sample size requirement.

## Results

The first results of model fit approached the expected requirements (χ^2^[362, N=271]=1124.93, *P*<.001, χ^2^/df=3.11, CFI=0.91, TLI=0.90, RMSEA=0.087, and SRMR=0.044), but remained insufficient due to the χ^2^/df value above 3 and the value of RMSEA above 0.08. Following the procedure of Kaplan [54] when the initial model did not provide an adequate fit to the data, we examined the modification indices suggested by the statistical software. The modification indices proposed that adding an error covariance between items 10, 11, and 12 of the Modified Falls Efficacy Scale would improve the model’s fit. This suggestion was theoretically possible and was used in the present study because these 3 items measured the same latent variable (fall-related self-efficacy). Subsequent analysis revealed that all of the fit statistics met the criteria for an acceptable fitting model, especially the RMSEA and the χ^2^/df, whose values were below the threshold of 0.08 and 3, respectively [50,51]: χ^2^(359, N=271)=988.95, *P*<.001, χ^2^/df=2.75, CFI=0.93, TLI=0.92, RMSEA=0.079, and SRMR =0.042.

**Figure 3:**
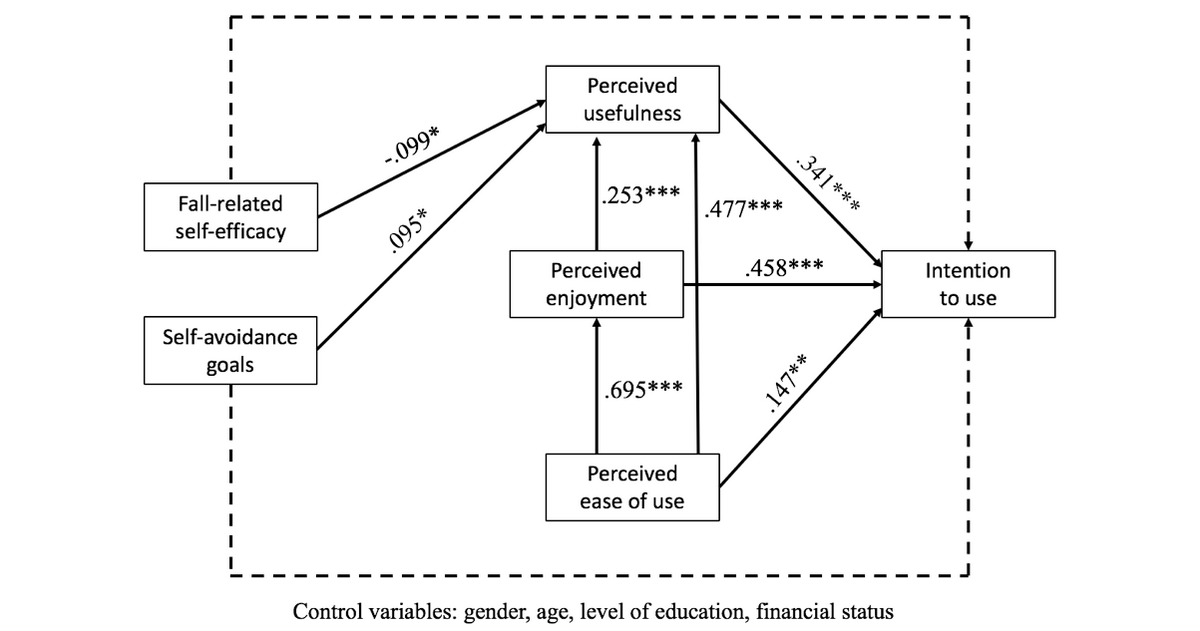
Validated structural model with standardized path coefficients. Dotted lines indicate nonsignificant paths. *P<.05, **P<.01, ***P<.001.

The results of the SEM analysis indicated that intention to use the VRH designed to reduce older adults’ risk of falling through its use in intervention programs was positively predicted by perceived usefulness (H1 supported; *P*<.001), perceived enjoyment (H2 supported; *P*<.001), and perceived ease of use (H3 supported; *P*=.01), but was not significantly predicted by fall-related self-efficacy (H9 not supported; *P*=.67) or self-avoidance goals (H10 not supported; *P*=.58). Perceived usefulness was positively predicted by perceived enjoyment (H4 supported; *P*<.001), perceived ease of use (H5 supported; *P*<.001), and self-avoidance goals (H8 supported; *P*=.03), and negatively predicted by fall-related self-efficacy (H7 supported; *P*=.03). Finally, perceived enjoyment was positively predicted by perceived ease of use (H6 supported; *P*<.001). All standardized path coefficients are presented in Figure 3.

Ancillary analyses showed that gender (women=–1, men=1) was a negative predictor of perceived usefulness (*P*=.04) and a positive predictor of perceived ease of use (*P*<.001). Age was found to be a negative predictor of both perceived enjoyment (*P*=.002) and perceived ease of use (*P*<.001). Finally, level of education was a positive predictor of perceived enjoyment (*P*=.04). These results are not represented in Figure 3 because they were not the most relevant ones relative to the main purposes of the study.

## Discussion

### Main Findings

Based on the TAM, the main purpose of this study was to examine acceptance among older adults of a VRH designed to prevent falls before its first use. The results provided interesting information. First, they highlighted that intention to use this device was positively predicted by perceived usefulness, perceived ease of use, and perceived enjoyment, which had been self-reported by older adults before using this VRH. These findings validate the TAM—for the first time to our knowledge—in the context of the intention to use a VRH to prevent falls through its use in intervention programs. Second, results also showed that fall-related self-efficacy and self-avoidance goals were negative and positive predictors, respectively, of the perceived usefulness of this VRH, providing external variables that had not yet been discussed in the TAM’s literature investigating this specific device.

The results of the SEM analysis showed that the most powerful predictor of intention to use the VRH was perceived enjoyment. High perceived enjoyment resulted in high intention to use the VRH. While perceived usefulness was the strongest predictor in most of the previous TAM studies [25,29], recent studies have highlighted that technologies based on virtual environments are considered hedonic technologies [55], and perceived enjoyment therefore becomes a strong predictor—possibly the strongest one [30]—of intention to use them. Perceived enjoyment was also recently found to be a relevant variable to take into account when studying older adults’ acceptance of VRHs [32,34]. Perceived usefulness also strongly positively predicted intention to use the VRH for preventing falls. This result was not surprising because (1) it was in line with the theoretical foundations of the TAM postulating that users tend to adopt a technology when they consider it useful [25,29], (2) it corresponds to the results commonly identified in the literature with various technologies, (3) perceived usefulness is a major variable for acceptance of utilitarian technologies [56], such as the VRH used for fall prevention in this study, (4) Peek et al [57] showed that the perceived usefulness of a technology identified by older adults is crucial for preimplementation acceptance (ie, when a technology has not yet been tried), and (5) perceived usefulness was already found to positively predict intention to use VRHs among adults [30] and older adults [32,34]. Finally, intention to use the VRH designed to prevent falls through its use in intervention programs was positively predicted by its perceived ease of use. When the VRH was perceived to be easy to use, older adults were more intent on using it. This result is consistent with the TAM, which postulates that users’ adoption of a technology depends on the level of difficulty associated with its use [25,29]. When the VRH used for fall prevention is considered simple and easy to understand by older adults, their intention to use it increases. While perceived ease of use was not the strongest predictor of intention to use this VRH in this study, it still remains a significant predictor that seems to be particularly relevant with older adults who are not necessarily familiar with such technologies [47]. In sum, this study validated, for the first time to our knowledge, the suitability of the TAM for investigating the acceptance of a VRH designed to prevent falls. Acceptance of the VRH is one of the conditions for the success of VRH-based training programs intended to prevent fall occurrence because if an older adult does not accept the device before using it, their likelihood of participating in the training program may decrease quite significantly [58]. Moreover, acceptance during the postimplementation stage (when users have already experienced a technology) may also be a condition of the success of technology-based training programs by increasing the motivational process necessary to maintain and improve participation throughout the program. However, studying acceptance after use was not a purpose of this study.

A second set of results showed that fall-related self-efficacy (ie, the perceived level of confidence of an individual when performing daily activities without falling) was a negative predictor of perceived usefulness of the VRH designed for fall prevention (ie, less confidence leads to more perceived usefulness). This result is not surprising because older adults who have a low fall-related self-efficacy develop a high fear of falling and reduce their activities [40]. Consequently, an intervention program using a VRH designed to prevent falls may seem more useful for older adults with a low fall-related self-efficacy than for those with a high self-efficacy, which explains the negative prediction found in this study. This is of particular interest in the context of health prevention because identifying fall-related self-efficacy may make it possible to propose such VRH-based interventions to older adults who probably need it most. Agreeing to participate in VR training programs would be a first step in helping to reduce the occurrence of falls and their negative consequences among older adults [37]. The results also showed that self-avoidance goals (ie, participating in a physical activity to avoid physical regression) were positive predictors of perceived usefulness of the VRH among older adults. The VRH designed to prevent falls through intervention programs based on physical exercise was found to be more useful to older adults who wanted to avoid physical regression because most of them aimed to maintain and preserve their current resources, especially in the physical domain [44]. The VRH can help them to do so in concrete terms. Finally, we also expected fall-related self-efficacy and self-avoidance goals to be direct predictors of intention to use the VRH. These hypotheses were not supported. Venkatesh [59] identified that in general external variables influence beliefs (perceived usefulness, perceived ease of use, and perceived enjoyment) rather than intention to use, which has been confirmed in this study. In sum, adding external variables (eg, fall-related self-efficacy and self-avoidance goals) as antecedents of perceived usefulness extends the TAM’s predictive power because these variables are selected to fit the technology (VRH in this study), context (fall prevention through intervention programs in this study), and users (older adults in this study) specifically investigated in studies about acceptance [32]. However, many other variables should also be examined as potential predictors of technology use by older adults, such as biophysical (eg, cognitive decline), psychological (eg, willingness to remain independent), and contextual (eg, financial means) factors [41,58].

### Current Limitations and Directions for Future Studies

This study had some limitations that might be addressed in future research. First, the participants’ responses were self-reported and may have been subject to social desirability [60]. Although the completion of the questionnaires was anonymous, older adults may not want to reveal that they are uncomfortable with new technologies. However, this risk remained limited in this study because the average scores for each variable of the TAM were in the middle of the scales and were not very high. Second, the external variables we chose for this study (fall-related self-efficacy and self-avoidance goals) were psychological rather than contextual variables. History of falls (ie, any fall event experienced by older adults during a specified period of time) may be a relevant variable to include in future studies because history of falls was shown to influence older adults’ behaviors in VR training [61]. Investigating other variables from the unified theory of acceptance and use of technology [26,36] might also be relevant to a better understanding of acceptance of the VRH used to prevent falls. For instance, the social influence of older adults’ companions and facilitating conditions (eg, having the necessary knowledge to use technologies and being able to get help) may be relevant variables to investigate, all the more so since a questionnaire in French has very recently been validated in young adults [21] and may be relatively easily adapted to older adults. Third, the psychological antecedents of the intention to use the VRH in this study were investigated without having experienced VRHs. Assessing older adults’ acceptance of this device after a first use would be interesting. Huygelier et al [33] have shown that older adults’ acceptance of VRHs increased after a first use lasting a few minutes compared with a control group. Accordingly, it can be hypothesized that the pattern would be similar for the VRH specifically designed for fall prevention. Finally, in future research conducted in our laboratory and elsewhere, the VRH will be used in long-term training programs (6 weeks at a rate of 3 to 4 sessions/week) to develop adaptive behavior of older adults in perfectly controlled, playful, and evolving contexts. A longitudinal study focusing on the evolution of acceptance throughout the intervention programs might provide relevant information about the dynamics of the acceptance process. Furthermore, long-term technology use by older adults may be influenced by disruptive forces that could cause changes in technology use, which might be investigated through longer longitudinal studies [57].

### Conclusions

This study focused on acceptance of a VRH designed for fall prevention through its use in training intervention programs as a possible moderator of the effectiveness of these devices. This study is a first step in this direction. Results showed that intention to use the VRH was predicted by its perceived usefulness, perceived enjoyment, and perceived ease of use. The results also suggested that perceived usefulness of the VRH was negatively predicted by older adults’ fall-related self-efficacy (ie, elderly individuals with less confidence found the VRH more useful) and positively predicted by their self-avoidance goals (ie, elderly persons who strived to avoid physical regression found the VRH more useful). Finally, our study allows better understanding of the factors that can influence older adults’ acceptance of a VRH designed to prevent falls.

## Abbreviations

CFI: comparative fit index
RMSEA: root mean square error of approximation
SEM: structural equation modeling
SRMR: standardized root mean square residual
TAM: technology acceptance model
TLI: Tucker-Lewis index
VR: virtual reality
VRH: virtual reality headset
